# MPZL1 forms a signalling complex with GRB2 adaptor and PTPN11 phosphatase in HER2-positive breast cancer cells

**DOI:** 10.1038/s41598-017-11876-9

**Published:** 2017-09-14

**Authors:** Alice Beigbeder, François J. M. Chartier, Nicolas Bisson

**Affiliations:** 1 Centre de recherche du Centre Hospitalier Universitaire (CHU) de Québec-Université Laval, Axe Oncologie, Québec, QC G1R 3S3 Canada; 2 Centre de recherche sur le cancer de l’Université Laval, Québec, QC G1R 3S3 Canada; 3 PROTEO-Quebec Network for Research on Protein Function, Engineering, and Applications, Québec, QC G1V 0A6 Canada; 4 Department of Molecular Biology, Medical Biochemistry and Pathology, Université Laval, Québec, QC G1V 0A6 Canada

## Abstract

HER2/ErbB2 is overexpressed in a significant fraction of breast tumours and is associated with a poor prognosis. The adaptor protein GRB2 interacts directly with activated HER2 and is sufficient to transmit oncogenic signals. However, the consequence of HER2 activation on global GRB2 signalling networks is poorly characterized. We performed GRB2 affinity purification combined with mass spectrometry analysis of associated proteins in a HER2+ breast cancer model to delineate GRB2-nucleated protein interaction networks. We report the identification of the transmembrane protein MPZL1 as a new GRB2-associated protein. Our data show that the PTPN11 tyrosine phosphatase acts as a scaffold to bridge the association between GRB2 and MPZL1 in a phosphotyrosine-dependent manner. We further demonstrate that the formation of this MPZL1-PTPN11-GRB2 complex is triggered by cell attachment to fibronectin. Thus, our data support the importance of this new signalling complex in the control of cell adhesion of HER2+ breast cancer cells, a key feature of the metastatic process.

## Introduction

Human Epidermal Growth Factor 2 (HER2) is an oncogenic receptor tyrosine kinase (RTK) belonging to the Epidermal Growth Factor (EGF) receptor family^1^. HER2 is overexpressed in 15–20% of breast cancer (BCa) patients (HER2+), and this is associated with poor prognosis^2^. HER2 overexpression initiates transformation and is also involved in metastasis as well as chemoresistance^3–5^. Targeted therapies such as trastuzumab were successfully developed and have been commonly utilized in the clinic for the past two decades^6, 7^. However, only one-third of patients with HER2-positive metastatic breast tumors will respond to this treatment^8, 9^.

Several molecular mechanisms detailing the consequences of HER2 activation have been highlighted^1, 3, 10, 11^. For example, the adaptor protein Growth factor Receptor-Bound protein 2 (GRB2), which is overexpressed in a number of BCa cell lines, was reported to be an essential proximal mediator of HER2^3, 4^. In addition, a constitutively active HER2 (Neu-NT) mutant that is unable to bind GRB2 displayed a lower oncogenic potential, arguing for a role for GRB2 in HER2 tumor-promoting functions^3, 4^. GRB2 bears a central Src-Homology (SH) 2 domain that can bind to phosphorylated residues within the pY-Φ-N-Φ consensus motif (where Φ represents an hydrophobic residue). This domain is required for binding to RTKs or cytoplasmic Tyr phosphorylated proteins and for the transmission of signals to downstream effectors^12, 13^. The latter process involves the two SH3 domains of GRB2, present on the N- and C-terminal side of the SH2 domain, respectively. Both GRB2 SH3 domains are able to bind Pro/Arg-rich motifs in a constitutive fashion, not requiring any posttranslational modification^14^. GRB2 key role is thus to couple signal from activated RTKs, such as HER2, to downstream effectors such as the RAS/MAPK activator SOS. GRB2 has previously been shown to be involved in BCa progression, *in vitro* as well as in murine models in which its expression level was correlated with tumor growth^15–17^.

MPZL1 (PZR) is a glycoprotein involved in extracellular matrix-induced signal transduction^18, 19^. It was recently demonstrated that HER2+ cell proliferation as well as resistance to targeted therapies are dependent on its expression levels^20^. MPZL1 contains a unique transmembrane domain and two intracellular Immunoreceptor Tyrosine-based Inhibitory Motifs (ITIM)^18^. Specific extracellular proteins such as fibronectin have been shown to promote SRC Family Kinases phosphorylation of MPZL1 ITIM-embedded Tyr241 and Tyr263 residues^19, 21, 22^. This latter event is required for the recruitment and subsequent activation of the PTPN11/SHP2 Tyr phosphatase^18^. In addition, phosphorylated MPZL1 was previously linked to the regulation of cell adhesion and migration^21, 23, 24^.

Evidences suggest that the organization of signalling networks is disrupted in cancer. In particular, phosphotyrosine (pTyr)-dependent networks are heavily modulated and even deregulated in cancer cells^25, 26^. The central role of GRB2 in HER2 signalling led us to hypothesize that the analysis of modifications within the GRB2 protein interaction network in HER2+ breast cancer cells would lead to the identification of key components required for downstream signalling. We combined GRB2 affinity purification (AP) with mass spectrometry (MS) identification of associated proteins in HER2+ breast cancer cells to delineate GRB2-nucleated protein interaction networks. We report the identification of MPZL1 as a new GRB2-associated protein. We show that MPZL1 associates with GRB2 in a pTyr-, PTPN11-dependent manner. Our data also demonstrate that the formation of MPZL1-PTPN11-GRB2 complex is regulated by cell adhesion on fibronectin.

## Results

### Protein interaction network mapping in HER2+ breast cancer cells reveals new GRB2 binding partners

**Figure 1:**
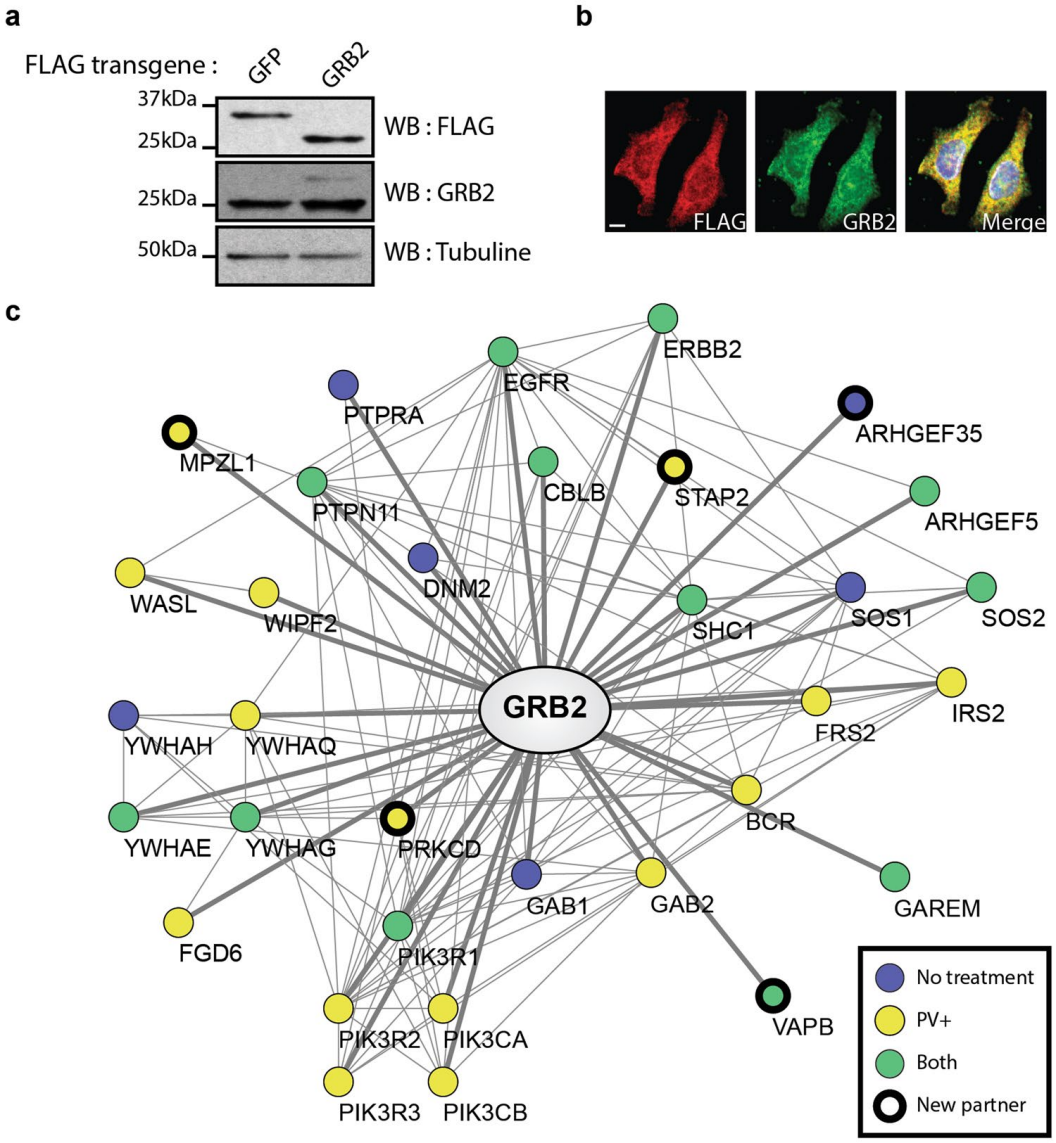
Protein interaction network mapping in HCC1954 HER2+ cells reveals new GRB2 binding partners. (**a**) Clonal stable cell lines expressing either 3xFLAG-GFP (30 kDa) or 3xFLAG-GRB2 (28 kDa) fusion proteins were analyzed by Western blot to evaluate transgene expression. (**b**) FLAG-GRB2 cells were analyzed by immunofluorescence to verify protein localization (scale bar = 10 μm). **(c)** GRB2 protein interaction network obtained by AP-MS from HCC1954 cells treated or not with a phosphatase inhibitor (PV). Proteins displaying a SAINT score above 0.9 are represented in the network (Supplementary Table S1). Edges represent interactions obtained by AP-MS (bold) or previously reported (narrow).

To characterize GRB2 signalling complexes in HER2+ breast cancer cells, we generated HCC1954 cell lines stably expressing 3xFLAG-tagged GFP and 3xFLAG-GRB2. We confirmed that the 3xFLAG-GRB2 chimera was expressed at sub-endogenous levels to prevent adverse effects on protein complex stoichiometry, and that it displayed the same cytoplasmic localization as endogenous GRB2 (Fig. 1a,b). Affinity purification (AP) of 3xFLAG-GRB2 from cell lysates followed by mass spectrometry (MS) identification led to the discovery of 18 proteins in GRB2 complexes, following background subtraction with the SAINT algorithm using 3xFLAG-GFP as a control (Fig. 1c, Supplementary Table S1)^27^. This list included a number of *bona fide* GRB2 SH2-dependent partners including PTPN11 and SHC1^28^, thus confirming the presence of pTyr residues resulting from HER2 overexpression and activation in HCC1954 cells. Nonetheless, in order to obtain the full complement of phospho-dependent interactions, we repeated experiments using sodium orthovanadate (pervanadate, PV), a phosphatase inhibitor that broadly elevates the total level of endogenous tyrosine phosphorylation. This led to the identification of another 12 GRB2-associated proteins (Fig. 1c, Supplementary Table S1). Among the 33 proteins that constitute the GRB2 signalling network in HER2+ cells, we discovered five candidates that were absent in the BioGRID database^29^. Remarkably, this list comprised three proteins that were previously suggested to play a role in breast cancer progression: the Rho family GTPases activating protein ARHGEF35^30^, the membrane-associated glycoprotein MPZL1^20^ and the adaptor protein STAP2^31^, confirming the validity of our approach.

### MPZL1 associates with GRB2 in a pTyr, PTPN11-dependent manner

**Figure 2:**
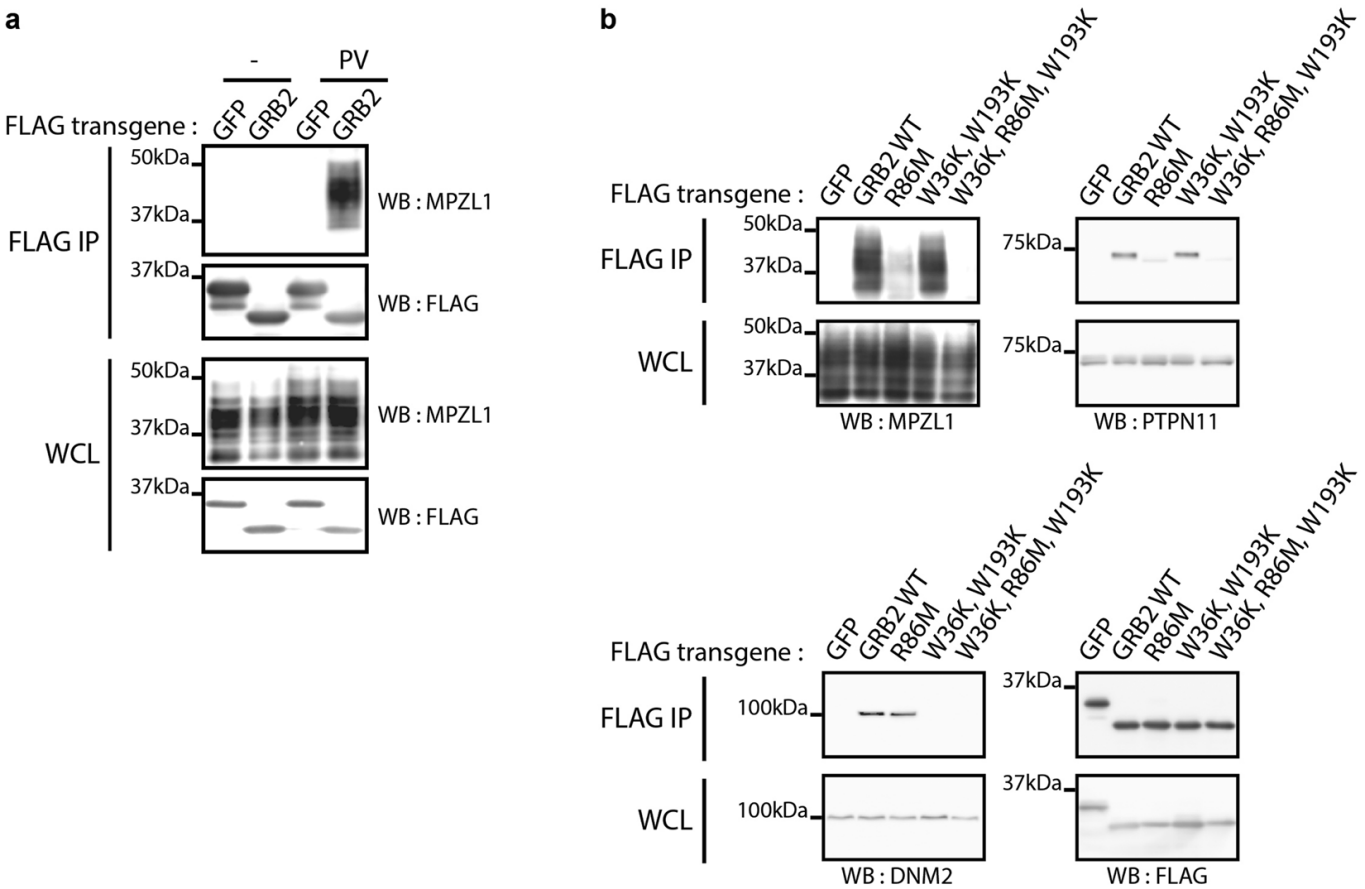
GRB2 associates with MPZL1 in a phosphotyrosine, SH2 domain-dependent manner. (**a**) HEK293T were transiently transfected with FLAG-GFP or GRB2 and lysates were used to perform an AP followed by Western blot analysis. (**b**) HEK293T were transiently transfected with FLAG-GFP or GRB2 (WT or point mutants), treated with PV and utilized as in (**a**).

To confirm the association between MPZL1 and GRB2 in other cell types, we transiently transfected 3xFLAG-GRB2 or a 3xFLAG-GFP control in HEK293T cells that we used for AP followed by Western blotting for MPZL1. We validated their association in cells treated with PV (Fig. 2a). To further characterize the interaction, we introduced substitutions that inactivate the conventional binding of the two SH3 domains (W36K, W193K) or the SH2 domain (R86M)^28^. Using *bona fide* GRB2 SH2 (PTPN11) and SH3 (DNM2) direct binding partners, we confirmed the specificity of the GRB2 mutants (Fig. 2b). We showed that the MPZL1 interaction was fully dependent on the presence of a functional SH2 domain (Fig. 2b). However, a survey of the MPZL1 sequence did not reveal consensus pTyr binding sites for GRB2 SH2 domain, arguing for an indirect interaction.

**Figure 3:**
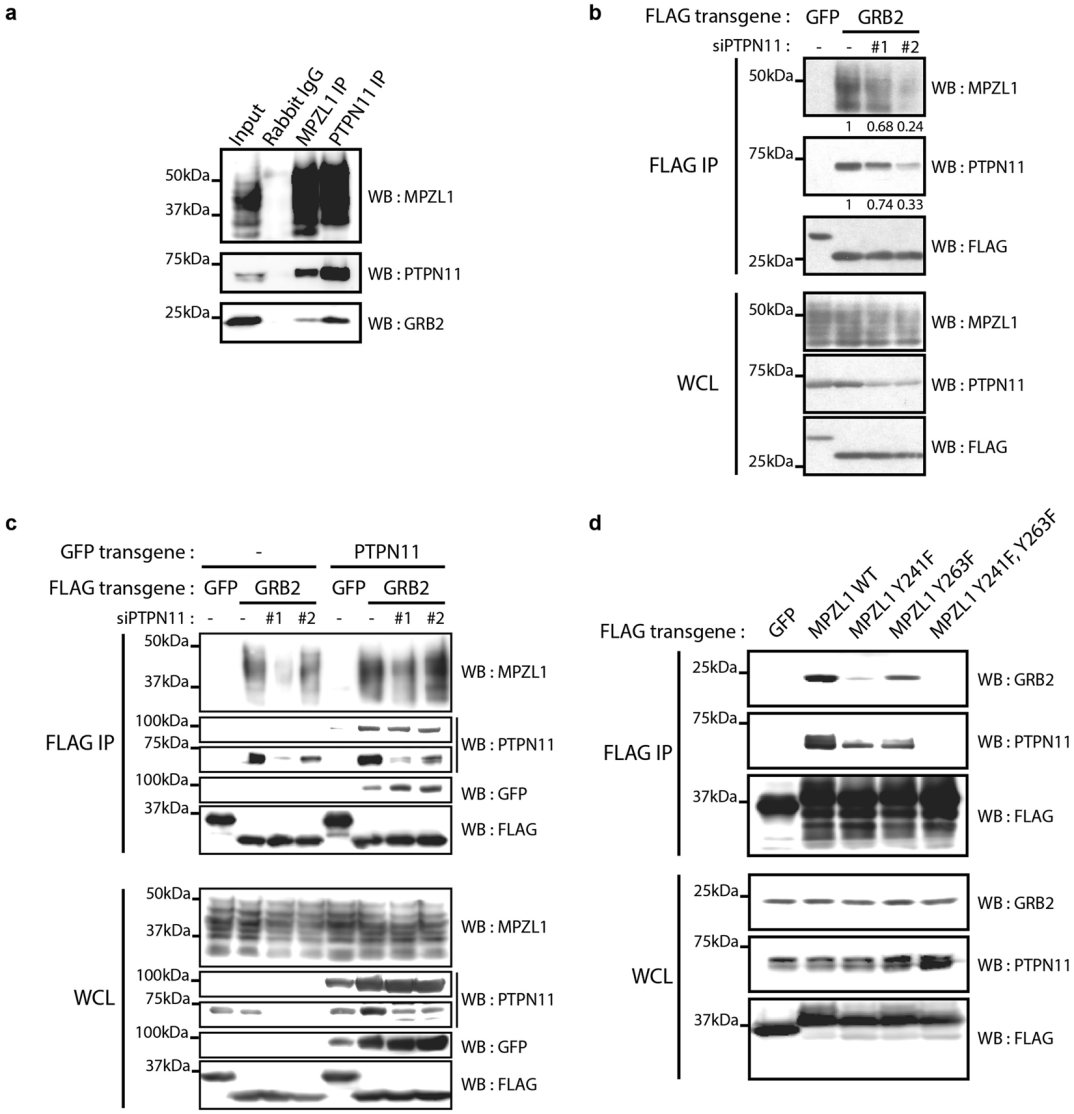
The tyrosine phosphatase PTPN11 is required as a scaffold for the GRB2/MPZL1 interaction. (**a**) HEK293T were treated with PV and lysates were used to perform APs using the indicated antibodies, followed by a Western blot analysis. (**b**) HEK293T cells were depleted of endogenous PTPN11 using DsiRNAs and transiently transfected with FLAG-GFP or GRB2 transgenes. FLAG APs were performed on cell lysates following PV treatment and analyzed by Western blot. **(c)** Cells obtained as in (**b**) were transfected with a GFP-tagged murine PTPN11 that is DsiRNA-resistant. Cells were treated with PV and FLAG APs were performed for each condition, prior to Western blotting analysis. (**d**) HEK293T cells were transfected with FLAG-GFP, MPZL1 (WT or non- phosphorylatable Y/F point mutants), treated with PV and utilized for FLAG APs - Western blotting.

The Tyr phosphatase PTPN11 is a well-characterized GRB2 SH2 direct binding partner (Fig. 2b) that was previously shown to associate with MPZL1 in a pTyr-dependent manner^18, 32^. We also identified PTPN11 in our AP-MS experiments from HCC1954 cells (Fig. 1c). To explore the existence of GRB2-MPZL1-PTPN11 signalling complex in intact cells, we immuno-precipitated endogenous MPZL1 and detected by Western blotting both PTPN11 and GRB2 (Fig. 3a). Conversely, MPZL1 and GRB2 were both present in immuno-precipitated endogenous PTPN11 complexes. These results confirmed that all three proteins were part of a pTyr-dependent protein complex. To assess the requirement of PTPN11 in the network, we examined the GRB2-MPZL1 association in cells transiently depleted of PTPN11 using DsiRNAs. We found that the presence of MPZL1 in GRB2 affinity-purified complexes was directly correlated with PTPN11 levels, suggesting that the latter is required as a scaffold for the signalling complex (Fig. 3b). This finding was further supported by our observation that MPZL1 levels were increased in GRB2 complexes upon expression of a GFP-PTPN11 transgene that is siRNA-resistant (Fig. 3c).

MPZL1 ITIMs (Tyr241 and Tyr263) were previously shown to bind both PTPN11 SH2 domains^32^. To determine whether the phosphorylation of these residues is required, we introduced Y241F and Y263F mutations, alone or in combination, in 3xFLAG-MPZL1. AP followed by Western blotting revealed that while PTPN11 binding was lost only for the compound MPZL1 mutant as expected, Tyr241 was mainly responsible to maintain the association with GRB2 (Fig. 3d). Together, our data show that MPZL1 associates with GRB2 in a complex that is dependent on tyrosine phosphorylation, using PTPN11 as a scaffold.

### MPZL1-PTPN11-GRB2 complex formation is regulated by cell adhesion on fibronectin

**Figure 4:**
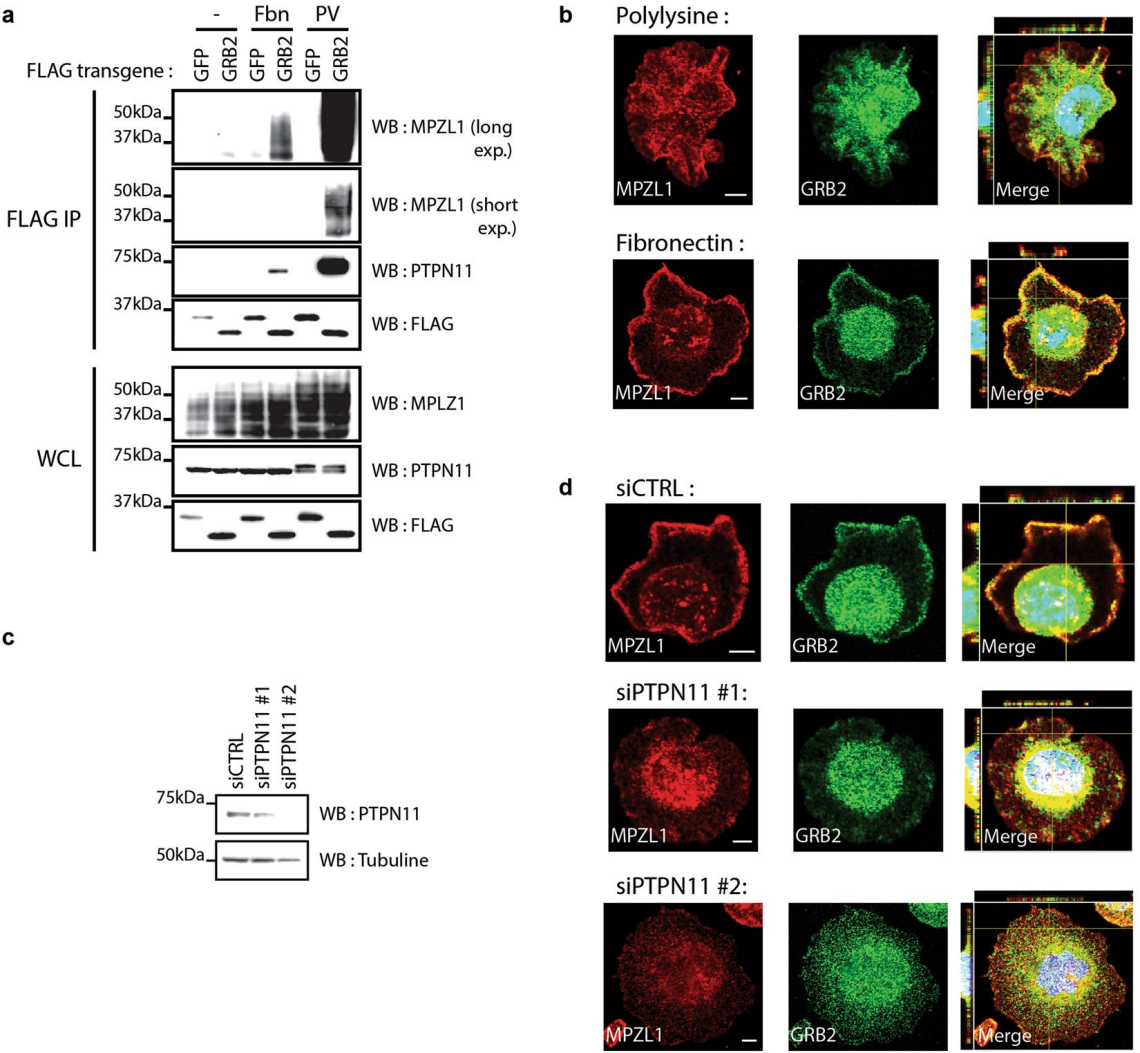
Cell adhesion on fibronectin triggers GRB2-MPZL1 complex formation at the plasma membrane. (**a**) HEK293T cells expressing FLAG-GFP or GRB2 were seeded on plastic or fibronectin for ten minutes before lysis. PV treated cells were used as a positive control. FLAG APs were performed for each condition and analyzed by Western blot as indicated. (**b**) HCC1954 cells were seeded on polylysine or fibronectin for 15 minutes before fixation. Immunofluorescence against GRB2 and MPZL1 was performed to assess the cellular localization. Confocal images of the *z*-axes (indicated by yellow lines) are shown on the left side (*y*-*z* axis) or on top (*x*-*z* axis) of the panels showing merged images, for which nuclei were stained with DAPI. Representative images are presented (scale bar = 5 μm). (**c**) HCC1954 cells were depleted of endogenous PTPN11 using DsiRNAs. (**d**) PTPN11-depleted cells were seeded on fibronectin for 15 minutes prior to fixation and immunofluorescence. Confocal images of the *z*-axes (indicated by yellow lines) are shown on the left side (*y*-*z* axis) or on top (*x*-*z* axis) of the panels showing merged images, for which nuclei were stained with DAPI. Representative images are shown (scale bar = 5 μm).

The MPZL1 membrane glycoprotein was shown to be phosphorylated by SRC family Tyr kinases upon its attachment to specific extracellular matrix proteins including fibronectin^19, 23^. To explore whether MPZL1-PTPN11-GRB2 complex formation is regulated by attachment to fibronectin, we expressed 3xFLAG-GRB2 or 3xFLAG-GFP in HEK293T cells, which we seeded either on plastic or on fibronectin. We analyzed FLAG affinity-purified complexes by Western blotting and found that both MPZL1 and PTPN11 were associated with GRB2 following fibronectin attachment (Fig. 4a). To extend this observation to HCC1954 cells, we assessed MPZL1 and GRB2 localization via immuno-fluorescence following attachment. MPZL1 and GRB2 were rather ubiquitously distributed in cells seeded on polylysine (Fig. 4b). In contrast, adhesion to fibronectin induced a colocalization of MPZL1 and GRB2 at the plasma membrane. Moreover, under this condition both proteins also localized in the nucleus; this was particularly evident for GRB2. To examine the requirement for PTPN11 in complex formation in HER2+ breast cancer cells, we analyzed GRB2 and MPZL1 localization in cells depleted of PTPN11 (Fig. 4c). We found that the proteins did not colocalize at the plasma membrane following PTPN11 knockdown when compared to controls (Fig. 4d). Together, our data indicate that the colocalization of GRB2 and MPZL1 at the plasma membrane in fibronectin-adhering cells depend on their ability to associate via PTPN11. Thus, our data along with previous findings lead us to propose a model where cell attachment to fibronectin induces the association between GRB2 and PTPN11, which are further recruited to MPZL1 at the plasma membrane to form a functional signalling complex.

## Discussion

We have used AP-MS to map a GRB2-centric protein interaction network in HER2+ breast cancer cells. We have identified GRB2 SH2-dependent interactors HER2, EGFR, PTPN11 and SHC1 in protein complexes via AP-MS from cultured HCC1954 cells, without stimulation or phosphatase inhibition. This observation confirmed that HER2 overexpression in these cells induces pTyr levels that are sufficient to nucleate GRB2 signalling complexes. This study and previous investigations on GRB2 signalling networks using quantitative mass spectrometry in HER2-negative cells clearly revealed that these SH2-dependent partners are usually not found in non-stimulated cells^28^ (Fig. 2a). Here, we found that HER2 overexpression was sufficient to initiate assembly of GRB2-centric protein interaction networks.

In addition to confirming a number of SH2- and SH3-dependent components of GRB2 signalling networks, our work revealed five new GRB2 binding partners. Among them, we found the membrane-associated glycoprotein MPZL1. Its association with GRB2 required either cell attachment on fibronectin, or treatment with a phosphatase inhibitor. This suggested that HER2 activation is not sufficient to induce MPZL1 phosphorylation on Tyr residues 241 and 263, which are required to sustain complex formation. This finding was surprising since it was previously reported that SRC activation may lead to MPZL1 phosphorylation^19, 23^. This activation of SRC was shown to happen either by a direct interaction with the cytoplasmic domain of integrins^33^, or in the context of HER2 overexpression^34^. Although it did not seem to be the case here, it remains possible that SRC regulates MPZL1 signalling. Consistent with this, it was described that MPZL1 stable overexpression led to SRC and SHP2 activation, the former being required for the regulation of migration and invasion of hepatocellular carcinoma cells dependent on cortactin^21^, which was also previously identified as a GRB2 binding partner^35^.

Interestingly, we have shown that the GRB2 association with MPZL1 relies predominantly on the phosphorylation of Tyr 241 on the latter, and less so on Tyr 263. This is contrary to reports suggesting that both residues are equally important for their association with PTPN11 SH2 domains^32^. Therefore, it remains possible that PTPN11 may not be the sole intermediate bridging GRB2 and MPZL1. Further experiments, including MPZL1 interactome mapping, will be required to identify putative additional components of this functional signalling complex.

In addition to MPZL1, a few other novel GRB2-associated proteins that we identified in our MS experiments were linked with breast cancer. The STAP2 adaptor contains an SH2-like domain. It is also a substrate for tyrosine kinase PTK6^36^, which is overexpressed in a significant fraction of breast tumors^37, 38^. It was reported that STAP2 depletion significantly decreases proliferation of breast cancer cells^39, 40^. The Rho guanine nucleotide exchange factor (GEF) ARHGEF35 (ARHGEF5-like) is another intriguing candidate due to its high similarity with ARHGEF5, which was also unambiguously identified in our analysis of GRB2 signalling networks (Fig. 1c). ARHGEF5 was previously shown to promote breast malignancy^41^. Both Rho GEFs are overexpressed in a significant proportion of breast tumors^38, 42^. STAP2 and ARHGEF35 represent two interesting candidates that might play important roles in GRB2-dependent HER2 oncogenic signalling.

Overall, our discovery that the MPZL1-PTPN11-GRB2 complex formation is regulated by cell adhesion on fibronectin is consistent with the implication of GRB2 in integrin signalling^43, 44^. This is also supported by the colocalization at the plasma membrane of GRB2 and MPZL1 upon cell adhesion on fibronectin (Fig. 4b). Further investigations will be required to establish the biological repercussions of the GRB2-MPZL1 association. The importance of MPZL1 for GRB2-PTPN11 localization in cells adhering to fibronectin, as well as its requirement for the activation of oncogenic signalling downstream of GRB2 in the context of HER2+ breast cancer are key questions that will need to be addressed.

## Methods

### Constructs

Human GRB2 (NCBI clone NM_002086) was subcloned into pMSCVpuro (Clontech) with an N-terminal 3xFLAG epitope tag (Sigma) to generate stable cell lines. The following cDNAs were subcloned into the pcDNA3.1- vector (Invitrogen) with a 3xFLAG or a GFP (Clontech) epitope tag: MPZL1 (BC007881), PTPN11 (Addgene #8381, Ben Neel). Point mutations were introduced using the QuickChange II strategy (Stratagene). All inserts were fully sequenced and protein expression was verified. The DsiRNA (IDT) sequences targeting PTPN11 were as follows (Seq#1: Sense: rCrGrCrUrArArGrArGrArArCrUrUrArArArCrUrUrUrCrAAA; Antisense: rUrUrUrGrArArArGrUrUrUrArArGrUrUrCrUrCrUrUrArGrCrGrUrA; Seq#2: Sense: rCrArArGrArA rCrArGrArCrGrCrArArGrAArArGrUrUrUAT; Antisense: rArUrArArArCrUrUrUrCrUrUrGrCrGrUrCrUrGrUrUrCrUrUrGrArU). Non-targeting DsiRNAs (IDT) were used as negative controls.

### Cell culture and transfection

HCC1954 and HEK293T cells were obtained from ATCC and cultured as recommended by the manufacturer. Transfections were performed using polyethylenimine (PEI) for HEK293T or AMAXA 4D nucleofection (Lonza) for HCC1954. Stable HCC1954 cells were selected with puromycin (0.75 μg/mL) for two weeks. Individual clones were picked and selected based on transgene expression. DsiRNAs (10 nM) were transfected using Jetprime (Polyplus) for HEK293T following manufacturer’s guidelines, or using Lipofectamine 2000 (Thermo Fisher) for HCC1954, as described^20^.

### Cell adhesion on fibronectin

Fibronectin 20 μg/mL (Sigma-Aldrich) or 0.01% polylysine (Sigma-Aldrich) solutions were used to coat petri dishes or glass coverslips for 1 hour at 37 °C. HEK293T or HCC1954 cells were resuspended with 0.2% trypsin (Invitrogen) for 1-5 minutes at 37 °C, pelleted and resuspended in serum-free medium. Cell solutions were incubated at 37 °C for 30 minutes with agitation, prior to seeding on fibronectin or polylysine-coated surfaces for 10 or 15 minutes before cell lysis or fixation, respectively.

### Cell lysis and affinity purification

Cells were grown to 95% confluency prior to lysis. Tyrosine phosphatase inhibition was achieved by addition of 100 μM sodium orthovanadate (Sigma-Aldrich) pre-activated with 30% H_2_O_2_ (1: 200) to cell medium for 20 minutes. Cells were washed with ice cold PBS before lysis. Proteins were extracted by scraping the cells in ice-cold lysis buffer, as described elsewhere^45^, and normalized according to their protein concentrations as measured by Lowry. Lysates were incubated for 1h30 at 4 °C with M2 affinity resin (Sigma-Aldrich) for FLAG APs. For endogenous APs, lysates were incubated with 5 μg of antibody overnight at 4 °C. Protein G-coupled Dynabeads® (Thermo) were then added and incubated for 30 minutes at 4 °C. Beads were washed three times with lysis buffer. For Western blotting, beads were resuspended in 4x Laemmli buffer. For MS experiments, beads were additionally washed twice with 20 mM Tris pH 7.4 and proteins were eluted by incubating with agitation at 4 °C with 50 mM H_3_PO_4_ before digestion.

### Western Blotting and antibodies

Proteins were resolved on 8 to 15% polyacrylamide gels and transferred onto nitrocellulose membranes (GE Healthcare). Loading was verified with Ponceau S (Sigma-Aldrich) staining. Membranes were blocked in 5% non fat milk in TBS for 1 hour at room temperature before being incubated with antibodies overnight at 4 °C in TBS-T supplemented with 5% BSA or 1% non fat milk according to manufacturer recommendations. Antibodies used were as follows: mouse GRB2 (BD Bioscience), rabbit PTPN11 (Santa Cruz), rabbit MPZL1 (Cell Signalling Technology), mouse tubuline (Cell Signalling Technology) or goat dynamin 2 (Santa Cruz). Membranes were washed with TBS-T before being incubated for 1 hour at room temperature with the following HRP-linked antibodies diluted in 1% milk in TBS-T: anti-FLAG M2 (Sigma-Aldrich), horse anti-mouse IgG (Cell Signalling Technology), goat anti-rabbit (Cell Signalling Technology) or rabbit anti-goat IgG (Thermo). Signal was revealed using BioRad Clarity Western ECL substrate and detected either on Hyblot CL autoradiography films (Denville) or with an Amersham Imager 600RGB (GE Healthcare). Signal quantification was performed using Image J software gel analysis tools (NIH).

### Immunofluorescence

Following cell adhesion, coverslips were washed twice with PBS and fixed with 4% paraformaldehyde pH 7.4 (BioShop) for 15 minutes at room temperature. After three washes with PBS, fixed cells were permeabilized using 0.2% Triton X-100 (Sigma-Aldrich) for 15 minutes at room temperature. Coverslips were blocked in PBS supplemented with 10% normal goat serum (Wisent) and 0.1% NP40 (Sigma-Aldrich) for 1 hour at room temperature and incubated with the following antibodies diluted in blocking solution: mouse FLAG (Sigma-Aldrich), mouse GRB2 (BD Biosciences), rabbit GRB2 (Santa Cruz) or rabbit MPZL1 (Cell Signalling Technology) for 1 hour at room temperature and then overnight at 4 °C. After washes in 0.1% NP40 in PBS, coverslips were incubated with Alexa 568-conjugated goat anti-rabbit (Invitrogen) or Alexa 488-conjugated goat anti-mouse (Cell Signalling Technology) antibodies for 1 hour at room temperature. They were washed three times with 0.1% NP40 in PBS and twice with PBS before being mounted on slides using ProLong Gold antifade with DAPI (Thermo Fisher). Pictures were acquired with an Olympus FV1000 using the FluoView software or with a Nikon Eclipse E600 imaging system using MetaView.

### Mass spectrometry sample preparation and analysis

Samples for mass spectrometry were prepared as detailed elsewhere^45^. Identification of the proteins was carried out on a 5600 + TripleTOF mass spectrometer (Sciex) with a nanoelectrospray ion source and coupled to a Ekspert NanoLC425 reversed-phase nanoscale capillary liquid chromatography (Eksigent). Digested peptides were separated on a nano cHiPLC column (3 u, 120 A C18, 15 cm × 0.075 mm internal diameter). Peptides were eluted in a 90-minute linear gradient of 5-35% of solvent B (acetonitrile, 0.1% formic acid) at 300 nL/min. Mass spectra were acquired using a data dependent acquisition mode using Analyst version 1.7 (Sciex). Each full scan mass spectrum (400 to 1250 m/z) was followed by collision-induced dissociation of the twenty most intense ions. Dynamic exclusion was set for a period of 12 seconds and a tolerance of 100 ppm.

MS/MS peak lists were generated using Protein Pilot version 5.0 (Sciex). MGF sample files were then analyzed using Mascot (Matrix Science, London, UK; version 2.4.0) and X! Tandem (The GPM, thegpm.org; version CYCLONE (2010.12.01.1). Both were set up to search against the Uniprot *Homo sapiens* reference proteome (March 2014 release, 69150 entries) assuming the digestion enzyme trypsin. The protein sequences of the GFP and GRB2 FLAG-tagged constructs were added to the database to estimate coverage. Databases were searched with a fragment ion mass tolerance of 0.100 Da and a parent ion tolerance of 0.100 Da. Carbamidomethylation of cysteine was specified as a fixed modification for all samples. Oxidation of methionine and phosphorylation of serine, threonine and tyrosine were specified as variable modifications for all samples. Conversion of glutamate and glutamine to pyroglutamate and deamidation of asparagine and glutamine were specified as variable modification for a subset of samples. Two missed cleavages were allowed. Scaffold version 4.7.5 (Proteome Software Inc.) was used to validate MS/MS based peptide and protein identifications. Protein identifications were accepted if they could be established at greater than 98% probability to achieve a FDR less than 1% and contained at least 1 identified peptide. Peptide and protein probabilities were assigned by the Protein Prophet algorithm^46, 47^. Proteins that contained similar peptides and could not be differentiated based on MS/MS analysis alone were grouped to satisfy the principles of parsimony.

Gene names for each of the validated proteins and their corresponding spectral counts in samples of each of three replicates were exported as a matrix according to SAINT algorithm guidelines^27^. SAINT express statistical analyses of the biological triplicates were performed with inclusion of the internal 3xFLAG-GFP controls, and proteins presenting a SAINT score above 0.9 were considered *bona fide* interactors. Each relationship between bait (GRB2) and prey (each validated protein) was represented in the final network with Cytoscape software (version 3.5.0)^48^. Known interactions between the proteins included in the GRB2 network were imported using the Cytoscape plugin tool Bisogenet^49^.

### Data availability statement

The datasets generated are available from the corresponding author on request.

## Electronic supplementary material

Dataset 1.

Supplementary Figure S1.
