# Supplementary material for: MPZL1 forms a signalling complex with GRB2 adaptor and PTPN11 phosphatase in HER2-positive breast cancer cells
Source: Sci Rep. 2017 Sep 14;7:11514. doi: 10.1038/s41598-017-11876-9 (PMC5599542; doi:10.1038/s41598-017-11876-9)

SUPPLEMENTARY FIGURE 1

MPZL1 forms a signalling complex with GRB2 adaptor and PTPN11 phosphatase in HER2-positive breast cancer cells.

Alice Beigbeder[1,2,3], François J. M. Chartier[1,2,3], Nicolas Bisson[1,2,3,4*]

[1] Centre de recherche du Centre Hospitalier Universitaire (CHU) de Québec-Université Laval, Axe Oncologie, Québec, QC G1R 3S3, Canada
[2] Centre de recherche sur le cancer de l'Université Laval, Québec, QC G1R 3S3, Canada
[3] PROTEO-Quebec Network for Research on Protein Function, Engineering, and Applications, Québec, QC G1V 0A6, Canada
[4]Department of Molecular Biology, Medical Biochemistry and Pathology, Université Laval, Québec, QC G1V 0A6, Canada.

* To whom correspondence should be addressed (nick.bisson@crchudequebec.ulava

Supplementary Figure S1

Original images from autoradiography films or Amersham imager used in the Western blots from figure 1 to figure 4. Areas used for the final figures are shown in a red rectangle. a- Images from Fig. 1a (lower quality images are shown here). b- Images from Fig. 2a. c- Images from Fig. 2b. d- Images from Fig. 3a. e- Images from Fig. 3b (lower quality images are shown here). f- Images from Fig. 3c. g- Images from Fig. 3d. h- Images from Fig. 4a. i- Images from Fig. 4c.

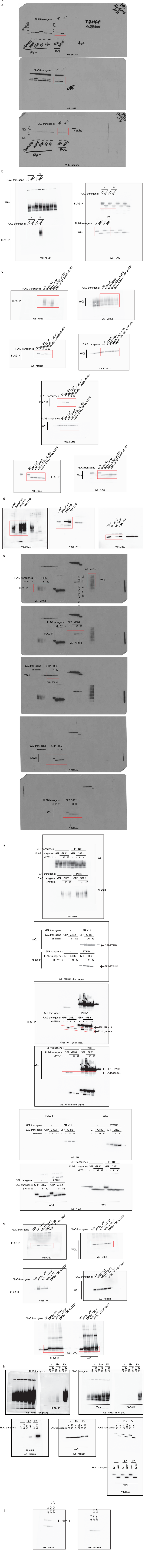

Supplement: Supplementary file 2 — Supplementary Figure S1. [file 41598_2017_11876_MOESM2_ESM.pdf]
